# It Is Time for Senescience

**DOI:** 10.3390/jcm11154542

**Published:** 2022-08-04

**Authors:** Francesco Mattace-Raso

**Affiliations:** Section of Geriatrics, Department of Internal Medicine, Erasmus MC University Medical Center, 3015 GD Rotterdam, The Netherlands; f.mattaceraso@erasmusmc.nl; Tel.: +31-10-7035979

Aging is the most impressive demographic phenomenon in human history. Due to fast medical developments, in addition to developments in the fields of transport, communication and economics, the world’s population is aging progressively and globally.

Humans have always reached extreme ages; it is not uncommon for historical individuals to have reached one hundred years of age. However, the pace of population aging today is much faster than in the past, and as a consequence of this, the percentage of persons reaching extreme longevity has increased beyond expectation. The number of persons aged 80 years or older is expected to triple worldwide in the next thirty years, with a projected 426 million octogenarians in 2050 [1].

As physicians, we will see a substantial increase in patients with chronic conditions, multimorbidity, and polypharmacy. Moreover, more and more patients will be faced with progressive functional and cognitive decline. Physicians have begun to encounter this novel category of patients: patients who were not included in our textbooks, and have only recently come to be described. The significant improvements in medicine in recent decades means that young adult patients have been able to survive diseases which were considered fatal in the past. Additionally, we have been able to prolong the prognosis of diseases which are still, unfortunately, fatal. In this way, we have ‘created’ a new category of patients who will undergo sophisticated mini-invasive and complex interventions, will use more and more medicines and, possibly, will have advantages with the support of technology.

Therefore, the study of age-related disorders is of paramount importance, and we must embrace a new concept: **SENESCIENCE**. This is a novel term to indicate the scientific approach of age-related physiology and disease.

This novel vision requires a novel approach from bench to bedside, and beyond. Only with a translational approach we will be able to understand and classify age-related disorders, unravel underlying mechanisms, discover new treatments and develop technology to adequately treat older patients and, when necessary, give adequate support in order to maintain independency and quality of life. Some of these topics have been treated in the Topical Collection “New Frontiers in Geriatric Diseases” of the *Journal of Clinical Medicine*.

 

**Evidence-Based Medicine and Not Eminence-Based Medicine**

There is a major need to expand and standardize medical guidelines for older patients. One of the pitfalls of geriatric medicine is that we miss, almost completely, the inclusion of older participants in clinical randomized control trials. This means that almost every medical treatment applied in older patients is an extrapolation of a treatment which has been shown to be effective and possibly harmless in young adults. Only recently have several large randomized control trials (RCT) included systematically older patients. The treatment of hypertension in older adults, for example, has been a matter of debate in recent decades. Is the treatment harmless and effective? What levels of blood pressure are recommended in persons aged >80 years? Recent RCTs have given answers to these questions. The HYVET trial showed, for the first time and conclusively, the benefits of blood pressure-lowering drug treatment in people aged 80 years or older [2]. Thereafter, other trials have investigated whether intensive blood-pressure-lowering intervention is safe and effective in older hypertensive patients. The SPRINT trial showed that an intensive blood pressure treatment results in significant cardiovascular benefit in high-risk patients with hypertension compared with routine blood pressure control [3]. Moreover, this study was the first trial on the treatment of hypertension stratifying for frailty. These studies, along with other RCTs, have provided high-quality knowledge which has been extremely useful to update (inter)national guidelines. Additionally, of course, we have to remember that treating old, frail patients will always require a tailored approach [4]. Finally, we cannot forget relevant topics when dealing with medications in frail patients: polypharmacy, drug–drugs interactions, and the balance between efficacy and safety [5,6,7].

 

**Stratification and Advanced Care Planning**

Information on prognosis is a necessity to optimize tailored treatments in older patients. It can be very challenging to establish an exact prognosis in persons with multimorbidities. Competing risk, drug interactions, reduced homeostasis and the risk of multiorgan failure, for example, are insidious enemies. Several attempts have been made to develop prediction tools. One of the most effective tools that has been produced in recent years is the multidimensional prognostic index (MPI) [8]. International and multicentric studies performed in different settings have shown that the MPI is useful to predict mortality and risk of hospitalization in community-dwelling and hospitalized older individuals. A multidimensional assessment of older people admitted to hospital may facilitate appropriate clinical and post-discharge management.

Evidence from these studies has prompted MPI_AGE Investigators to formulate recommendations for healthcare providers, policy makers and the general population, which may help to improve the cost-effectiveness of appropriate healthcare interventions for older patients [9]. The application of this tool in specific categories of patients has shown that the MPI can be a useful tool to assess frailty and predict which patient will have a higher chance of benefiting from a TAVI procedure [10]. Moreover, also in heterogeneous populations, such as patients suffering from cognitive decline, the MPI has been able to predict mortality. These findings need to be confirmed in larger and even more heterogeneous populations of patients with cognitive decline. If confirmed, the MPI could be used as a novel tool for risk stratification and medical decisions in this specific category of patients with a high need for tailored support [11].

 

**From Bench to Bedside**

Finally, to better understand age-related disease, it is necessary to understand age-related cellular mechanisms, DNA repair, and the degradation of tissue. In vitro investigations are strongly needed. A recent experimental study found that alterations in the extracellular matrix where the muscle spindles are embedded could help to partly explain the peripheral mechanisms underlying age-related decline in functional changes [12]. The knowledge acquired by these approaches will determine future developments.

The last concept with must consider is domotics: a technology which can be used by older individuals experiencing functional and/or cognitive decline to assist in controlling devices or events in their environment. The recent developments in this field are promising, and have given much inspiration to gerotechnology.

In conclusion, aging is here to stay, and we must grasp the opportunities offered by this unknown phenomenon in order to grapple with the future of older patients.
